# Intersections of Gender and Cognition in Older Adults

**DOI:** 10.1093/geroni/igaa057.2440

**Published:** 2020-12-16

**Authors:** Shana Stites, Jason Flatt, Carol Derby

## Abstract

The National Institutes of Health (NIH) is committed to supporting rigorous science that advances what is understood about the influences of sex and gender in health and disease in order to inform the development of prevention strategies and treatment interventions. In research on aging and Alzheimer’s disease, sex/gender disparities in key outcomes are common. But, much of this research hinges on asking a single question: Is the patient or research participant male or female, man or woman? This practice offers few options for disambiguating sociocultural effects associated with gender from those related to biologic sex. It also assumes that self-reports are a suitable proxy for social phenotypes and that a dichotomous variable adequately captures the wide-range of sociocultural effects attributable to gender. The premise of this symposium is to evaluate how gender interacts with cognitive outcomes in order to advance measurement. This symposium will review evidence from five distinct lines of research on associations between gender and cognition for individuals and for individual’s interactions with their family members: (1) effects of normative shifts in American education on cognition in older adults; (2) hospitalization as a risk factor for cognitive decline in racially diverse American men and women; (3) caregivers who identify as sexual and gender minorities (SGM or LGBTQ+) and care for persons with dementia; (4) correlates of cognitive function in SGM older adults; and (5) differences in adults’ cognition based on childhood exposure to women’s social empowerment in 30+ Organisation for Economic Co-operation and Development (OECD) countries.

